# An Innovative Place-Based, Neighborhood-Level Approach to Address Health Disparities in Medically Underserved Areas of Memphis, TN

**DOI:** 10.1007/s40615-025-02357-1

**Published:** 2025-03-05

**Authors:** Alexandria M. Boykins, Alana J. Schilthuis, Hannah D. Thomas, Deborah Ogunsanmi, Satya Surbhi, Susan W. Butterworth, Susi L. Suttle, Colbie E. Andrews, James E. Bailey

**Affiliations:** 1 Tennessee Population Health Consortium, University of Tennessee Health Science Center, 956 Court Ave., Coleman D228, Memphis, TN USA; 2 Center for Health System Improvement, College of Medicine, University of Tennessee Health Science Center, Memphis, TN USA; 3 Division of General Internal Medicine, Department of Medicine, University of Tennessee Health Science Center, Memphis, TN USA; 4 Department of Preventive Medicine, University of Tennessee Health Science Center, Memphis, USA

**Keywords:** Health coaching, Medically underserved area, Primary care, Access, Preventive care, Healthcare delivery, Community health centers, Obesity, Diabetes, Community health workers

## Abstract

Little data demonstrates the feasibility of place-based, neighborhood-level care delivered by health coaches in medically underserved neighborhoods to expand access to essential primary care and address health disparities. This concurrent mixed-methods pilot study describes experience with the innovative Neighborhood Health Hub Program in Memphis, TN. Patient characteristics, including body mass index (BMI), blood glucose, blood pressure, and service utilization, were assessed. Key informant interviews and community meetings were conducted in an initial community listening period to guide program development. Patient experience with program services was assessed using semi-structured client interviews. In year 1, 355 year-one clients were outreached, 146 (41.1%) through community events, 149 (42%) walk-ins, 38 (10.7%) door-to-door communication, 34 (9.6%) telephone, and 9 (2.5%) referral. Of the 198 (56.1%) fully screened, mean age was 52.0 (± 15.9) years, 94.5% were African American, 55.8% female, and 32.7% without a primary care provider. Baseline blood pressure was uncontrolled (≥ 140/90) in 52.3%, BMI was ≥ 30 in 50%, and random plasma glucose was high (≥ 130 mg/dl) in 23.4%. The majority (68.3%) participated in individual health coaching. Sixty-eight group sessions had an average of 4 participants (range 1–13) and were focused on chronic illness management (39.7%), exercise (26.5%), or nutrition (25.0%). Major qualitative themes highlighted the importance of social barriers and social support for chronic condition management. Place-based, neighborhood-level care delivered by health coaches in medically underserved neighborhoods is a promising approach for extending primary care, expanding access to essential preventive and primary care, reducing health disparities, and improving patient outcomes.

## Introduction

Obesity, hypertension, and diabetes mellitus are among the most common chronic conditions among adults in Tennessee [1, 2]. The Mid-South region of the USA is home to some of the largest health disparities in the nation due to various socio-demographic factors and health behaviors that contribute to high rates of chronic disease [3–5]. As defined by the Centers for Disease Prevention and Control (CDC), “social determinants of health (SDoH) are the nonmedical factors” (such as economic hardship, neighborhood, and access to care) “that influence health outcomes” [6] and contribute to racial and ethnic health disparities [7, 8]. SDoH are strongly associated with incidence of chronic health conditions including hypertension and diabetes mellitus [9, 10], variations in screening rates for these conditions [11, 12], and adverse health outcomes [13–16]. Numerous studies conclude that addressing SDoH is paramount to improving health outcomes [17–20]. In low-income, medically underserved areas of metropolitan areas like Memphis, TN, facing shortages of primary care providers [21], approaches are needed to extend primary care into the neighborhoods with the greatest social risks and health disparities using lay health workers.

Memphis, TN, is the largest majority Black/African American major metropolitan area in the USA, and while its residents experience very high rates of adverse SDoH and associated chronic illness, access to essential primary and preventive care is extremely limited, especially in majority Black neighborhoods. All predominantly Black neighborhoods in Memphis are classified as primary care shortage areas and medically underserved areas [22]. Previous studies have also linked poor healthcare access and adverse health outcomes to historic racial discrimination, including the practice of redlining Black neighborhoods for residential and business loans resulting in major disparities in the ability to build wealth, compounding geographic and racial inequities [23–26]. In 2023, almost a third (27.1%) of Black Memphians were living in poverty, a 3.8% increase since 2019 and a much higher rate (8.9%) compared to their white counterparts in the city [27]. These numbers are even higher in communities like the “Uptown-Memphis” area where the Black poverty rate is 77.4%, ranking the area fifth in the city for overall poverty [27]. As a result of all these factors, racial health disparities in Memphis are profound [28], and innovative approaches are needed to address the complex interplay of race, racism, and SDoH underlying disparities at the neighborhood level.

The utilization of health coaches and other types of community health workers (CHW) in the USA continues to increase as their benefits are demonstrated [29–31]. CHW are often members of the community that share similar ethnicity, cultural values, and lived experiences as the patients they serve with a unique ability to provide culturally appropriate care [32]. Known benefits of CHW services include improved access to healthcare, increased utilization of essential population health screening services, improved adherence to health recommendations, increased communication between community members and healthcare providers, increased trust in the healthcare system, and reduced need for emergency and specialty services [30, 32–36]. Previous research has demonstrated the effectiveness of health coaches and other types of CHW in improving control of multiple chronic illnesses [37–39]. However, little evidence is available regarding the feasibility of deploying health coaches onsite in medically underserved neighborhoods as an extension of primary care. Health coaches have the potential to deliver low-cost, place-based, neighborhood-level care; help to address transportation barriers to healthcare; and expand access to essential primary and preventive care.

Research has shown that health coaches can play a critical role in screening, identification, and “closing the loop” on social needs related to SDoH [40]. Health coaches support team-based person-centered care for chronic disease and lifestyle-related conditions by engaging patients in self-management and treatment adherence, assisting patient navigation of the healthcare environment to improve access to essential care, and providing referrals or resources to address social determinants of health (SDOH) impacting health and access to care. Health coaches are typically recruited from the community served to foster communication and trust [41] and receive competency-based training in motivational interviewing, the best-proven counseling approach for empowering health behavior change [42–44]. Health coaches address SDoH by: actively evoking and listening to patients about their social environment; understanding and taking into account how factors like income, housing, education, and access to healthcare are impacting their health and well-being; and connecting them to relevant community resources and support networks to mitigate these challenges [40]. Health coaches enhance skills such as problem-solving and encourage resiliency, boosting self-efficacy and a sense of agency to commit to lifestyle changes despite negative social risks. Essentially, health coaches act as a bridge between their patients and the services that can improve their overall well-being beyond just following their treatment plan.

Research has demonstrated the advantages of place-based interventions for addressing SDoH, improving population health, and reducing health inequalities [45], but the best community settings for these interventions remain unclear. Community health centers have been shown to help bridge gaps in care, reduce disparities, and positively impact health outcomes but face challenges related to available health professional manpower, focus on acute illness care, lack of community engagement and trust, and low levels of geographic penetration of highest-need neighborhoods that limit their ability to close gaps in needed essential preventive care [46, 47]. For example, a meta-analysis of 114 diabetes self-management systemic reviews highlighted the need to “invest efforts in strengthening social support and innovative community care approaches” as well as incorporating the SDoH to effectively care for patients [48]. Other studies focused on caring for patients with multiple chronic conditions in medically underserved areas found that the traditional healthcare system commonly leaves patients with fragmented care and needs persist for additional support in managing a healthy lifestyle including education and self-management education [49, 50]. Previous studies on the healthcare experiences of underserved populations concluded that quality care needed to be culturally relevant, address perceived barriers, and enhance coping strategies [51]. Preliminary experience in other communities seeking to extend primary care into the community using CHW suggests that this approach can help uncomplicate the medical system, increase self-efficacy, and strengthen traditional primary care by providing additional support for adherence and self-management [36, 37, 52–55]. These studies suggest that employing CHW, using peer counseling, and supporting self-management can substantially strengthen the primary care safety net in medically underserved neighborhoods.

This study aims to describe the first-year experience delivering care through the Neighborhood Health Hub Program in Memphis, TN, at the University of Tennessee Health Science Center (UTHSC) Health Hub–Uptown. This paper aims to assess the feasibility of this innovative approach for delivering place-based, neighborhood-level care by health coaches in medically underserved neighborhoods. We sought to assess whether such a program could effectively recruit and retain people with unmet essential healthcare needs by evaluating the characteristics of the population served and the services they utilized. Furthermore, we aimed to assess the patient experience of care through the NHH Program, including changes or impacts on overall access to care, self-efficacy, and overall health, and explore the underlying mechanisms through which NHH services drive participant engagement, behavior change, and improvements in health outcomes from the client’s perspective. We hypothesized that the NHH Program will prove feasible and will significantly reduce barriers to care for people in medically underserved communities with limited access to primary care.

## Methods

This concurrent mixed-methods pilot study describes the first-year experience with the Neighborhood Health Hub Program, an innovative approach for place-based, neighborhood-level care delivered by health coaches in free-standing health hubs located in underserved neighborhoods of Memphis, TN. Patient characteristics, services utilized, and body mass index, blood pressure, and blood glucose level were assessed on intake and at each visit using the Research Electronic Data Capture (REDCap) tool hosted by the UTHSC.

### Study Setting and Participant Population

The study population included all adult clients seen at the UTHSC Health Hub–Uptown in its first year of operation. The Uptown neighborhood of Memphis, TN (ZIP Code 38,105), has substantial poverty levels (39.3%) and is predominantly African American (78.2%) [56].

### Intervention

The Neighborhood Health Hub Program opened its first neighborhood health hub in the Uptown neighborhood of North Memphis, TN, on November 1, 2021, to begin a community listening period and get community input on specific services most needed and desired by neighborhood residents. UTHSC Health Hub–Uptown staff began seeing clients on January 3, 2022.

The Neighborhood Health Hub employs UTHSC-certified health coaches to provide screening services and counseling to clients in both individual and group settings [57, 58]. The Health Hub also provides community activities such as fitness and cooking classes. Upon first introduction to the Health Hub, clients are offered screening for obesity, diabetes mellitus, and hypertension and are connected to an individual health coach. We provide a comprehensive health coaching and client navigation program to address SDoH. The health coaches provide a system of support in weight management, blood pressure control, and glycemic control by encouraging healthy eating strategies, physical activity regimens, medication adherence, social support, and tobacco cessation. The Health Hub facilitates access to essential primary care and mental health services. Our initiative also addresses social risks and the behavioral causes of obesity, hypertension, and diabetes that increase risk of poor health outcomes in general, as well as complications from COVID-19.

UTHSC Health Hub–Uptown faculty and administrative leadership kept logs of key events, activities, and community input throughout program development and Hub opening on November 1, 2021, through the end of the first full year of operation on December 2022. This information was used to document care processes, cultural tailoring of intervention components, and major lessons learned.

### Design

The overall study design is concurrent mixed methods based on the National Institute on Minority Health and Health Disparities theoretical framework [59]. We chose this framework and research approach to facilitate understanding the breadth of client real-world experience related to all of the domains of social determinants of health as well as potential biological and healthcare system factors impacting that experience at the individual, interpersonal, community, and societal levels.

### Quantitative Analysis

Quantitative data on primary outcomes, including enrollment, type of service utilization, and baseline patient characteristics, were collected for all patients attending the UTHSC Health Hub–Uptown in its first year of operation from January through December 2022. Descriptive statistics were employed to assess patient characteristics, frequencies, and means for utilization of services. Pre-post analysis was performed to assess metrics on weight, blood sugar, and blood pressure control for patients with at least 1 year of follow-up visits.

### Qualitative Analysis

The qualitative portion of the study employed the phenomenological method. We completed semi-structured client interviews and a concurrent demographic survey over 3 weeks in July of 2023. Health Hub clients served as key informants for this study. Purposive criterion sampling was used to recruit participants over 18 years of age, with two or more visits to the UTHSC Health Hub–Uptown, one or more chronic conditions, and the ability to read, write, and speak English. Potential participants were recruited using flyers at the Health Hub, and health coaches and a student researcher were present to provide study information to potentially eligible patients. Following a brief survey administered before each interview to collect demographic information, including age, gender, race, ethnicity, and education levels, a student researcher conducted semi-structured interviews lasting 30–40 min. Interviews were conducted without a health coach present to organically elucidate patient stories and experiences. Questions focused on the participants’ journey and experience with their chronic condition(s) before and after seeking care through the NHH Program, including changes or impacts on overall access to care, self-efficacy, and overall health. Interview data was collected using audio recordings and researcher field notes on any significant body language, facial expressions, or otherwise relevant data not captured through sound. Interviews were conducted in person and over Zoom depending on participant preference and availability. All data was kept anonymous, and participants received a $25 gift card as compensation for their time.

Recordings of the interviews were transcribed verbatim, and the transcripts underwent thematic analysis to identify emerging codes and themes analyzed through the qualitative software, NVivo. A phenomenological approach that focused on the lived experiences of participants was used to identify and evaluate the common themes that emerged. To minimize bias and ensure reliability, two independent coders were involved to achieve intercoder agreement [60]. Moreover, field notes were examined for thematic patterns and combined with the data from key informant interviews. To ensure consistency and agreement between the analysts, a meeting was held to review and reconcile any discrepancies. A comprehensive codebook was generated, comprising a list of codes and their corresponding descriptions.

Demographic data obtained through surveys underwent analysis using appropriate statistical software. Categorical variables were analyzed and reported as frequencies and percentages.

## Results

### Community Engagement

Two months before beginning service delivery at the UTHSC Health Hub–Uptown, Hub staff and leadership began a community listening period to engage residents and community leaders and gain their expertise and guidance on the greatest community health needs and potential services to be provided. This process started with a highly publicized community meeting at the nearby Community Center in which community input was formally requested and provided. This was followed by monthly lunch and learn events hosted at the hub facility hosted by the Executive Dean of the College of Medicine, at which ongoing guidance and input were obtained from resident experts and community leaders. These events typically included 5 to 10 guests as well as health hub staff.

Key lessons learned during the listening period included the identification of strong resident and client interest in support groups for healthy living, weight loss, diabetes, tobacco cessation, and increasing physical activity; health coaching; group exercise activities; managing high blood pressure; health activities for children; COVID testing and vaccination; assistance with health insurance, nutrition, housing, transportation, and employment; and primary and specialty care referrals. Numerous clients shared stories of empowerment and increased health efficacy through health coaching (Table 1).

**Table 1:** De-identified participant story captured from community-engaged listening sessions

Ms. Jane Doe was a 55-year-old woman with borderline diabetes who experienced:
*Frustration* with her primary care provider (PCP) for not listening and being rude
Her PCP *prescribing her too much medication*; wanting to immediately start her on Metformin without sufficient explanation
*Loss of trust* in the healthcare system; she did not want to continue seeing any doctor Ms. Doe participated in several health coaching and support group sessions over 4 weeks. Health
Hub staff assisted her with scheduling a visit with a different PCP. As a result, she now reports changing her diet, losing weight, and regaining trust in the medical profession

### Client Characteristics and Service Utilization

**Fig. 1:**
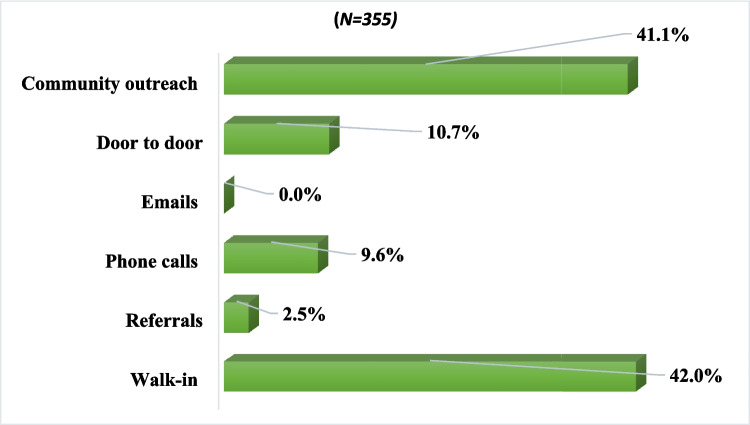
Methods of initial contact for all clients expressing an interest in neighborhood health hub services

Between January and December 2022, a total of 355 clients visited the Health Hub, of which 198 (56.1%) were screened and completed their intake form. Figure 1 shows the methods of initial contact for all clients. Of the 355 clients prescreened, 146 (41.1%) were reached via a community outreach program, 149 (42%) of our clients walked into the health hub, 38 (10.7%) were reached by door-to-door communication, 34 (9.6%) by phone calls, and 9 (2.5%) by referrals.

Table 2 shows the baseline characteristics of the 198 clients screened at the Health Hub. The mean age was 52.0 (± 15.9) years with most clients being African Americans (94.5%) and females (55.3%). Additionally, 67.3% of our clients reported having a primary care provider, 62.9% receiving a COVID-19 vaccination, and 92.7% reported that they were not worried about losing their housing in the next 2 months. Among the 198 screened patients, 47.7% had a blood pressure < 140/90, 25.6% had a blood pressure < 130/80, 50% had a BMI ≥ 30, 23.4% had high random plasma glucose (≥ 130 mg/dl), and 5.0% had high random plasma glucose (≥ 200 mg/dl).

**Table 2:** Baseline characteristics of the population served

	*n* = 198
Demographic characteristics	
Age^a^, years, mean (SD)	52.3 (15.9)
Gender, *n* (%) Female Male	111 (55.8) 88 (44.2)
Race, *n* (%) African American White Some other race/mixed race Prefer not to answer	188 (94.5) 6 (3.0) 4 (2.0) 1 (0.5)
Ethnicity^b^, *n* (%) Hispanic or Latinx Non-Hispanic or Non-Latinx Prefer not to answer	2 (1.0) 195 (98.5) 1 (0.5)
Insurance type^c^, *n* (%) Medicare Medicaid/Tenncare Commercial type insurance Uninsured/self-pay Other Unknown	34 (17.1) 27 (13.6) 59 (29.7) 27 (13.6) 10 (5.0) 6 (3.0)
Primary care provider^d^, *n* (%) Yes No	101 (67.3) 49 (32.7)
Clinical characteristics	
Weight^e^, pounds, mean (SD)	194.5 (50.1)
Body mass index (BMI)^e^, kg/m^2^, mean (SD)	31.4 (8.4)
Systolic blood pressure (SBP)^f^, mmHg, mean (SD)	133.6 (20.7)
Diastolic blood pressure (DBP)^f^, mmHg, mean (SD)	83.0 (12.3)
Random blood glucose^g^, mg/dl, mean (SD)	124.2 (52.6)
Blood pressure ≤ 130/80, *n* (%)	51 (25.6)
Blood pressure ≤ 140/90, *n* (%)	95 (47.7)
BMI ≥ 30, *n* (%)	88 (50.0)
Random blood glucose ≥ 130 mg/dl, *n* (%)	37 (23.4%)
Random blood glucose ≥ 200 mg/dl, *n* (%)	8 (5.0%)
Covid vaccination status^h^, *n* (%) Not vaccinated Vaccinated (2 doses) Vaccinated (2 doses + booster)	19 (9.6) 48 (24.1) 47 (23.6)
Social determinants of health	
Housing insecurity^i^, *n* (% yes)	11 (7.3)

^a^Missing age value, n = 12

^b^Missing ethnicity value, n = 1

^c^Clients can have more than one insurance type

^d^Missing primary care provider value = 49

^e^Missing height, weight, and BMI value = 23

^f^Missing SBP and DBP values = 25

^g^Missing random blood glucose value = 40

^h^Missing covid vaccination status value = 48

^i^Missing worried about losing housing value = 48. Clients were asked, “Are you worried about losing housing in the next two months”

**Fig. 2:**
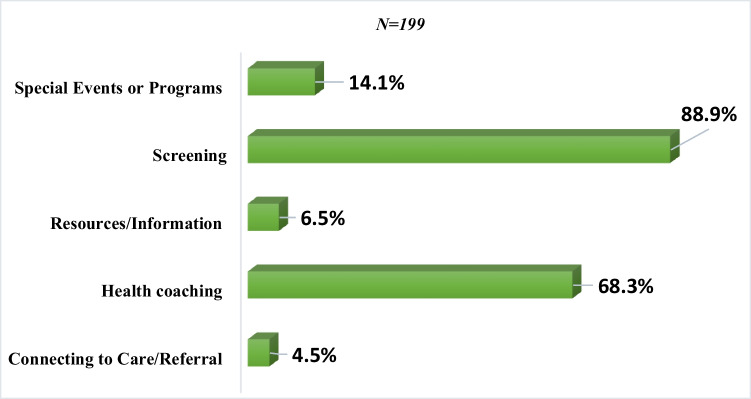
Services provided to clients*. *Percentages do not add to 100% because clients could receive more than one type of service

**Fig. 3:**
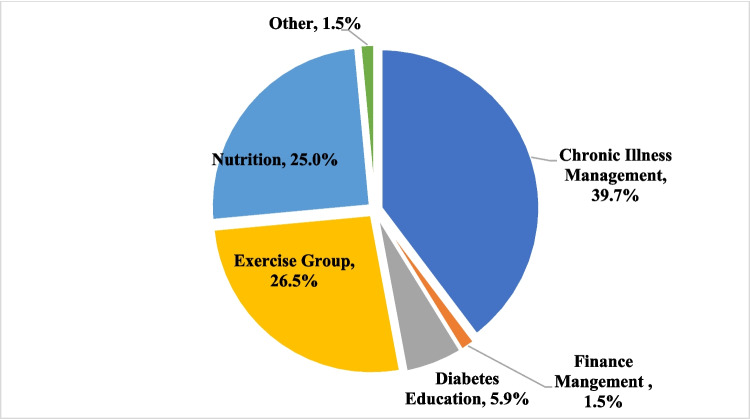
Group sessions (*N* = 68)

Of the 198 screened patients, the services that were provided to the clients included overall health screening (88.9%), health coaching (68.3%), special events or programs (14.1%), resources/information (6.5%), or connecting to care (4.5%) (Fig. 2). A total of 68 group sessions were conducted in 2022 (Fig. 3). On average, each group session had 4 participants, with a minimum of 1 and a maximum of 13 participants. The majority of the group sessions were focused on chronic condition management (39.7%), exercise (26.5%), and nutrition (25.0%). Other focus areas included diabetes education (5.9%) and finance management (1.5%).

### Client Experience

For the clients who participated in the qualitative portion of the study (*N* = 14), the median age was 66.5 years, 93% were Black/African American, 71% female, 43% were single/never married, and 28.6% had a bachelor’s degree. The majority (64%) received their main income from Social Security and 43% had other retirement income. Of note, 71% had access to transportation by car with 43% reporting a transportation time to the hub of 15 min or less. The 14 participants had a high prevalence of chronic conditions with 64% reporting diagnosis of hypertension, 50% high cholesterol, 50% arthritis, and 29% diabetes.

#### Social Barriers to Chronic Condition Management

The first major theme that emerged was the key role of social barriers to chronic condition management related to SDoH. Key subthemes included (1) provider competency, (2) quality of care, (3) neighborhood and physical environment, (4) economic stability, and (5) food security. Clients suggested that a range of social barriers made it difficult to manage their chronic health condition(s), prompting them to access the NHH Program. Key informants indicated that provider competency was a barrier to good communication and positive patient-provider relationships. Clients explained that the lack of competency increased their anxiety and decreased their trust. Clients often reported better experiences with providers who shared their gender, race, cultural background, or illness. Similarly, key informants struggled with the quality of care they received. Participants mentioned receiving a lower quality of care when their questions and concerns were ignored and there was little to no explanation and education about their condition. Further, participants were frustrated when medication was presented as the only option with no advice on lifestyle changes. Key informants who experienced poor patient-provider communication used health coaches to find a new primary care provider and to increase their understanding of health information and behavior change treatment options. In addition, clients experienced increased burdens when trying to manage and prevent chronic illness while navigating daily life barriers. Key informants described struggling with social factors like transportation access, limited income for healthy food options, difficulty accessing disability and social security benefits, or even having a safe neighborhood to exercise. Clients often shared these barriers with their coaches and other hub staff in hopes of finding solutions and connections to social service and community-based resources.

#### Social Support for Chronic Condition Management

Social support for chronic condition management emerged as the major theme related to client experience with the NHH Program, while the type of support received was coded as a sub-theme. Major subthemes included (1) psychosocial support, (2) empowerment, and (3) tangible benefits. Participants remarked on the ways their confidence benefited from coach affirmations; they gained awareness from information supplied during hub chronic illness classes and were aided by emotional support provided by coaches and “classmates.” Empowerment also emerged as a main theme and was an important strength of the model. Key informant interviews indicated that patients were empowered by professional, encouraging, and engaged health coaches. Another important theme was tangible benefits. A key advantage of the NHH Program was its provision of tangible benefits to patients that filled in the gaps left by SDoH, including diabetes and nutrition education, free cost, access to health screenings, connections to care, supplemental groceries, and assistance obtaining housing and navigating federal benefit applications (Table 3).

**Table 3:** Selected themes by area and illustrative quotes from semi-structured client interviews

Themes	Illustrative quotes
Social barriers to chronic condition management	
Provider competency	“That’s what I’m talking about. Somebody who actually knows and deals with it themselves. Cause I feel like before, I’m not saying, you got to deal with the disease yourself to figure out and know you know anything about it. But it was helpful talking to somebody who understood my challenges, so that when I’m talking to the doctor, or whatever it’s not like oh, they’re just writing it down, but they’re not really hearing you.” “Representation, going to providers who I feel like can kind of understand what I’m going through or understand my experience in the healthcare industry with healthcare professionals, I tend to like to go to people who I can uh identify with in one way or another.”
Quality of care	“And I just feel like I’m not getting the answers that I should get as a patient. All I know is they want me to all these medications.” “I just feel like I’ve been going to doctors back-to-back. But nothing is getting better every time I go. My blood pressure is high.” “I’m gonna figure it out. Nobody is helping me. So I just started talking to people start going online and started reading stuff that you know, just doing the best I could to try to get things under control.” “You want me on this diet? Put it in paper. Write what I can and can’t do. I mean, I’m doing my part. But there should be another part. If you want the weight off, I feel like there should be a way to help me get it off.”
Neighborhood and physical environment Economic stability Food security	“You have to pay to exercise. I don’t have no income. I can’t walk there. And now you got to realize you can’t walk in the neighborhood you live in, because there’s so much going on. You want to go somewhere where you’re safe and comfortable to work out.” “I have Fresh Market down the road and Kroger’s down the road. So yeah, I can get to it. It’s just expensive.” “I’ve been trying to get my disability, but nobody is trying to help me. If you get approved for disability, then they have those transportation services that could take you to a community center or anywhere you wanted to work out.” “Those food drives where they come with a bunch of cans, and some loaves of bread. High sodium, high carbs. I’m trying to stay away from all that. Not everybody has real access to *real* healthy food.”
Social support for chronic condition management	
Psychosocial support	“Every time it looked like I was losing hope, it looked like they knew it. And they were like, oh, let me do it this way. Let’s try it this way. You know? And I was like, how did you know? They must have seen it.” “Well, I have my like personal hype person. So, every time I come in hearing the *Okay, I see you*. That’s helpful. I love that.” “The people that were in class with me. We just decided to try to keep in touch with one another to hold each one of us accountable.” “You feel like they really care when you go over there [at the Health Hub]” “What helped me was understanding how high blood pressure is a silent killer if we don’t do something about it. I want to live. I want to know for me and my grandchildren. I’m doing research, looking at the information on the Internet, reading and participating in the classes at the health hub.”
Empowerment	“I changed my eating habits, everything changed. I’m trying to equip myself to manage my own health. I’m not as fearful about it.” “Being in control. True enough, I have diabetes, but diabetes don’t have me. And before I started coming here, diabetes had me, but I’ve got diabetes now.”
Tangible benefits	“I still had a lot of the recipes in a book that I could try, and then, when we were there for the classes, after we cooked the same meal, they gave us the food [groceries] to go home and make it.” “I learned how to you know how to deal with diabetes as far as helping family members that are diabetic. What to cook, what not to cook. How to decrease certain things, and then going through the cooking class. It helped me prepare healthier meals, to be intentional about drinking enough water, and portion sizes. So going through both of those classes and networking with the people that I met has been life changing.” “I like the fact that after I can go over there and get my blood pressure checked. And you know and kind of talk to the people to see what’s going on with my health.” “People were very inviting. Made you feel like you’re not really at a hospital setting or anything and you’re waited on right away.”
Future directions	
Service expansion	“I guess the purpose of the health hub is not actually a direct service provider. I just thought it would be good to periodically have one there.” “I don’t know if they have any kind of support groups, but I think something bringing the people together, sitting down talking, kind of sharing would be helpful.” “I think mental health counseling. A lot of people just don’t know how to open up and so they are living in silence.”
Outreach	“More awareness. I don’t think a lot of people know that it’s there. I certainly didn’t know about it before the meeting. You can reach out to more people in the community.” “It could be outreach for kids because we got kids that’s got health problems” “Put the word out in the community more about the health hub being here. Because in that community there’s probably quite a few people that could benefit. I mean even knocking on some doors. Some church doors, or something. I would just like to see more people get more benefits out of it, because I know I’m benefiting from it.”

#### Future Directions

Two major themes emerged concerning future directions for the Health Hub: (1) expansion of services and (2) increased community outreach. Participants expressed a desire for service expansion at the Health Hub. Participants frequently mentioned the importance of emotional and mental health, suggesting the Health Hub should offer individual mental health counseling and group support. While understanding the uniqueness of the NHH Program, participants recommended offering limited primary care since the Health Hub was an accessible and affordable resource in the community. Participants described the ways they “discovered” the hub, often through word of mouth or by accident. They advocated for increasing community outreach through media promotion and connections with local organizations and places of worship. In addition, they believed it was important to promote to all ages using a whole-family approach since adolescents were also experiencing health issues. Key informants explained that community outreach was necessary to raise awareness of health hub resources and benefits.

## Discussion

This study provides preliminary evidence of the feasibility of place-based, neighborhood-level care delivered by health coaches in medically underserved neighborhoods to expand access to essential primary care. We found that clients were most successfully engaged through one-on-one connections, community outreach events, and walk-ins. First-year utilization data demonstrates a strong demand for screening and individual and group health coaching services by medically underserved neighborhood residents at very high risk of complications from chronic conditions. We found obesity and uncontrolled hypertension in over half of clients seen, consistent with demonstrated high chronic disease burden in low-income areas of the Mid-South [3–5]. Furthermore, in the targeted high-need neighborhood, we found that one-third of clients did not have a primary care provider, consistent with documented shortages of primary care providers in medically underserved areas in the Mid-South [21]. This pilot data suggests that the neighborhood health hub approach may be a feasible place-based preventive care model for other medically underserved high-need communities in the USA.

Our qualitative findings demonstrate the effectiveness and value of the neighborhood health hub approach from the perspective of community residents. Health hub clients commonly reported overall high value of health hub services and experiences of increased social support, enhanced motivation, and empowerment in their care. Clients indicated that the Health Hub filled critical gaps in care created by SDoH by providing multiple forms of social support for chronic condition management, including appraisal [61], emotional, tangible, and informational [62, 63]. The provision of social support by health coaches and fellow support group participants was perceived as a key facilitator of client engagement, empowerment, and disease self-management. By fostering trust and rapport with hub clients, health coaches were able to help patients tap into their motivation for change, build a deeper knowledge of health information relevant to their health needs, increase their self-efficacy and self-determination through positive affirmation, and build skills in collaborative problem-solving. In addition, clients had access to tangible support (e.g., transportation assistance, care referrals, social aid assistance, and food access). Further, clients reported that Health Hub services filled critical gaps in primary and preventive care access, helping to increase their awareness related to diet, nutrition, fitness, and weight loss and assisting them in making positive behavior changes. These qualitative findings affirm the value of place-based, neighborhood-level care from the patient perspective.

The findings of this study are consistent with previous studies showing that health coaches deployed in high-need community locations can effectively improve access to essential primary and preventive care. The current study aligns with previous research demonstrating that these lay health workers can improve utilization of key population health screening services, increase communication between community members and healthcare providers, and reduce the need for emergency and specialty services [30–36]. Furthermore, our early dissemination and implementation experience provides real-world validation of previous research demonstrating the effectiveness of health coaches and other types of CHW in improving control of chronic illness [37–39]. Our qualitative findings are also consistent with previous research indicating enhanced patient engagement and trust in the healthcare system when lay health workers are employed who share similar ethnicity, cultural values, and lived experiences as the patients they serve [32].

Furthermore, this preliminary experience suggests that the NHH approach is feasible and can help close gaps in needed essential preventive care which community health centers are challenged to address because of a lack of sufficient health professional manpower and limited geographic penetration of highest-need neighborhoods [46–50]. The NHH approach offers a promising opportunity for improvement in “strengthening social support and innovative community care” and addressing SDoH as suggested by a recent meta-analysis [48]. Furthermore, the NHH approach has the potential to directly address the needs for culturally relevant self-management education and support for a healthy lifestyle [49–55]. Thus, our experience suggests that the NHH approach for using health coaches to provide peer counseling and support for self-management has the potential to substantially strengthen the primary care safety net in medically underserved neighborhoods.

The current pilot study is subject to some limitations. First, this early dissemination and implementation study is limited in size and, while it does provide an initial assessment of the feasibility of the neighborhood health hub approach, it does not assess long-term effectiveness or sustainability. Further studies are needed to assess the approach’s real-world effectiveness and sustainability by expanding the approach to additional medically underserved neighborhoods and implementing the submission of billing codes for health coaching services. In addition, the current study assessed the place-based, neighborhood-level preventive care in a single location, potentially limiting study generalizability. In addition, our qualitative data showed that the program identified some key areas for future improvement from the patient perspective, including desires for expanded service delivery in the areas of mental health, primary care services, and increased physical activity opportunities.

## Conclusions

This study provides early evidence of the innovative NHH approach in Memphis, TN, regarding the feasibility and utility of deploying health coaches in medically underserved neighborhoods as an extension of primary care. This early experience shows that non-clinical staff, such as health coaches, can deliver low-cost, place-based, neighborhood-level care that can help to address fundamental barriers to essential healthcare related to SDoH and expand access to essential primary and preventive care. Further studies are needed to evaluate the effectiveness and long-term sustainability of the neighborhood health hub approach. Thus, the current study demonstrates the feasibility of an innovative place-based model for investment in neighborhood assets and personnel to strengthen social support and build communities of health where they are needed most.

## Data Availability

The data that support the findings of this study are available on request from the corresponding author, JEB. The data are not publicly available due to restrictions (e.g., data contain information that could compromise the privacy of research participants.
